# Astrocytic clasmatodendrosis in the cerebral cortex of methamphetamine abusers

**DOI:** 10.1080/20961790.2017.1280890

**Published:** 2017-01-31

**Authors:** Zhiyong Zhang, Qingjin Gong, Xueying Feng, Dongchuan Zhang, Li Quan

**Affiliations:** a Department of Forensic Pathology, Zhongshan School of Medicine, Sun Yat-Sen University, Guangzhou, China; b Forensic Science Center, Shunde Branch of Foshan Public Security Bureau, Foshan, China; c Shanghai Key Laboratory of Crime Scene Evidence, Shanghai Institute of Forensic Science, Shanghai, China

**Keywords:** Forensic science, forensic pathology, immunohistochemistry, methamphetamine, human brain, astrocyte, clasmatodendrosis

## Abstract

Postmortem investigation of methamphetamine (MA) abuse is an important task in forensic pathology. The present study investigated morphological changes in the astrocytes in the parietal cerebral cortex of MA abusers. Glial fibrillary acidic protein immunoreactivity in the cerebral cortex was examined in forensic autopsy cases for MA-detected group and control group. Clasmatodendrotic astrocytes (including those with swollen cell bodies and disintegrating distal processes) were frequently observed in the cerebral cortex of MA abusers. Quantitative analysis using a colour image processor showed a concomitant increase in the astrocyte area and astrocyte-to-vessel area ratio (size and number of astrocytes) in the grey matter in acute MA fatality and other MA-involved cases, although the astrocyte area (size) was also increased in cases of asphyxiation. The total astrocyte area (size) in the white matter was significantly higher in MA fatalities and asphyxia than in the other groups involving MA abusers. Those indices were independent of blood MA level, age, sex, survival or postmortem time. These observations suggest the increasing number and hypertrophic changes of astrocytes in the grey matter in MA abusers can be the outcome of long-term abuse, while disintegrating distal processes may exist only in acute fatal MA intoxication.

## Introduction

Drug abuse is a worldwide social problem related to crime, traffic and non-traffic accidents and physical and psychological hazards. Abuse of the illegal psychostimulant methamphetamine (MA) has become an international public health problem with an estimated 15–16 million users worldwide, making MA the second most widely abused drug after cannabis [1]. MA can lead to physical impairments, psychological damage and even death [2,3]. In such cases, postmortem diagnosis of the cause of death and the influence of the drug is an important task for the forensic pathologist [4]. However, it may be difficult, in particular, in those cases involving other diseases [5–8] or drug combinations [9].

Acute MA dosing causes a variety of physical and mental disorders including restlessness, confusion, anxiety, hallucinations, cardiac arrhythmias, hypertensive crises, hyperthermia, metabolic acidosis, circulatory collapse, convulsions and coma. Both short-term and long-term serial and parallel toxic processes may cause neuronal metabolic deterioration involving an increase in oxidative stress, which can be enhanced by hyperthermia, and finally result in apoptosis and neuronal necrosis [10–15].

Moreover, chronic MA abuse can increase tolerance to the drug, and the acute toxic effects may not clearly depend on the dose [16–18]. A previous study suggested that the number of glial cells, which have been shown to synthesize and release neurotrophic factors, may be increased in relation to MA doses [19]. However, low doses of MA can protect dopaminergic neurons against larger oxidative stress injury [20] or reduce the level of neurotensin in the basal ganglia [21,22], and the precise mechanisms of MA-induced neuroprotection remain incompletely understood, with little being known regarding the molecular mechanisms involved.

The glial reactions in the striatum of chronic MA users who did not abstain from MA use and died of drug intoxication have been reported [23]. In the present study, astrocytic morphological changes in the cerebral cortex of MA abusers were quantitatively analysed to investigate the neuropathological effects of MA abuse in humans.

## Materials and methods

### Materials

Formalin-fixed paraffin-embedded brain tissue specimens of medicolegal autopsy cases (*n* = 56) at Zhongshan School of Medicine, Sun Yat-Sen University, and Medical School, Osaka City University, were examined. Causes of death were classified into MA-detected group (*n* = 25), including acute MA fatality subgroup (*n* = 15) and other deaths of MA abusers subgroup (*n* = 10) (polytrauma from fall, *n* = 5; hanging, *n* = 1; aspiration, *n* = 1; drowning from fresh water, *n* = 2; spontaneous cerebral haemorrhage, *n* = 1), and control group (*n* = 31), including four subgroups (asphyxia, *n* = 7; acute injury death, *n* = 9; fire death, *n* = 9; acute myocardial infarction, *n* = 6). Relevant patient data, including age and sex, were included in Table 1. In this study, to eliminate any subjective causal explanations, we categorized the fatalities involving MA on the basis of the immediate cause of death clearly accountable from pathological and toxicological evidence as follows: fatal MA intoxication, deaths from the other extrinsic causes (traumas) and strokes caused by natural diseases. Fatal MA intoxication was defined as death due to the drug (blood MA level: 0.03–13.10 μmol/dL, median, 1.28 μmol/dL) without any evidence of other fatal pathologies. Deaths from other extrinsic causes/traumas were defined as showing an apparent fatal injury or definite evidence of asphyxiation or drowning irrespective of the blood MA level (0.05–8.37 μmol/dL, median, 1.42 μmol/dL). Deaths due to stroke from a natural disease were defined as showing evidence of an acute fatal disease (spontaneous cerebral haemorrhage) irrespective of the blood MA level (5.63 μmol/dL). In control cases, the acute myocardial infarction group consisted of those that showed macro- and microscopic findings of acute ischemic heart diseases without any evidence of cause of death other than cardiac arrest [24–27].

**Table 1.** Case profile.

Cause of death	Case number	Male/female	Age/years (median)	Survival time (h, median)	Postmortem interval (h, median)
MA-detected group (*n* = 25)					
MA intoxication^a^	15	13/2	20–48 (33.5)	0.5–30 (5.5)	8–48 (25.0)
MA-related death^b^	10	7/3	20–67 (42.5)	<0.5–3 (0.5)	9–48 (30.0)
Control group (*n* = 31)					
Mechanical asphyxia	7	3/4	23–58 (40.0)	<0.5	11–32 (20.0)
Acute injury death^c^	9	7/2	26–71 (55.0)	<0.5–18 (3.5)	9–42 (20.8)
Fire fatality^d^	9	8/1	50–82 (60.5)	<0.5 (0.5)	8–23 (18.0)
Myocardial infarction	6	5/1	58–78 (63.5)	<0.5–2 (0.5)	7–28 (21.0)
	
Total	56	43/13	20–82 (46.5)	<0.5–30 (1.5)	7–48 (19.2)

a Blood MA level: 0.03–13.10 μmol/dL (median, 1.28 μmol/dL).

b Polytrauma from fall (*n* = 5), hanging (*n* = 1), aspiration (*n* = 1), drowning from fresh water (*n* = 2) and spontaneous cerebral haemorrhage (*n* = 1). Blood MA level: 0.05–8.37 μmol/dL (median, 1.42 μmol/dL).

c Blunt injury: chest (*n* = 2) and head (*n* = 6); sharp instrument injury: chest (*n* = 1).

d Carboxyhemoglobin (COHb) level: 56%–98%.

MA, Methamphetamine.

### Methods

#### Tissue sections

Serial sections (5-μm-thick) were prepared from the brain tissue specimens of the parietal lobe (precentral gyrus) for haematoxylin-eosin (HE) staining and immunostaining of glial fibrillary acidic protein (GFAP).

#### Immunostaining

Rabbit polyclonal anti-GFAP antibody (1:1000; Santa Cruz biotechnology, Inc., USA) was used, with a 3-h incubation at 37 °C, on a Vectastain Universal Elite ABC kit (Vector Laboratories, Burlingame, CA) according to the manufacturer's instructions (counterstained with haematoxylin). Endogenous peroxidase was inactivated by incubation with 3% hydrogen peroxide for 5 min. For the control study to confirm the specificity of immunostaining, phosphate buffered saline (PBS) or normal rabbit serum was substituted for the primary antibody.

#### Quantitation of the astrocyte areas

GFAP-immunopositive astrocytes in the cerebral cortex were quantitatively analysed using a colour image processor (IPAP, Sumica Technos, Japan). The total astrocyte area ratio (%) and astrocyte-to-capillary vessel area ratio (%) in the grey matter and the total astrocyte area ratio (%) in the white matter were estimated [28].

#### Chemical analysis

Blood alcohol was determined using headspace-gas chromatography/mass spectrometry [29]. Drug analyses were performed by gas chromatography/mass spectrometry.

#### Statistical analyses

Regression analysis was used to examine the relationship of the total astrocyte area and astrocyte-to-vessel area ratios (%) with the ages of the patients and their blood MA levels. Comparisons between groups were performed using the Student's *t*-test and the Mann–Whitney *U*-test. The logistic regression and stepwise regression tests were used in the multivariate analyses. These analyses were performed using Microsoft Excel (Microsoft Corporation, Redmond, WA) and Statview (version 5.0; SAS Institute, Cary, NC). *P* values less than 0.05 were considered statistically significant.

## Results

**Figure 1.**
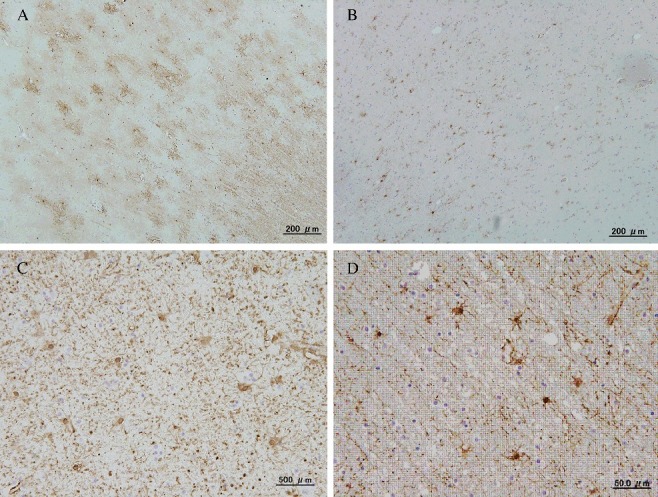
Immunostaining of glial fibrillary acidic protein (GFAP) in the parietal cerebral cortex: (A, C) Hypertrophic changes and number increased in the astrocytes in an acute methamphetamine (MA) fatality (blood MA concentration: 2.71 μmol/dL, 45-year-old man, 28 h postmortem); (B, D) A control case showed no astrocyte changes (a fire fatality, blood carboxyhemoglobin concentration: 78.5%, 65-year-old man, 18 h postmortem). (A, B) grey matter and (C, D) white matter.

**Figure 2.**
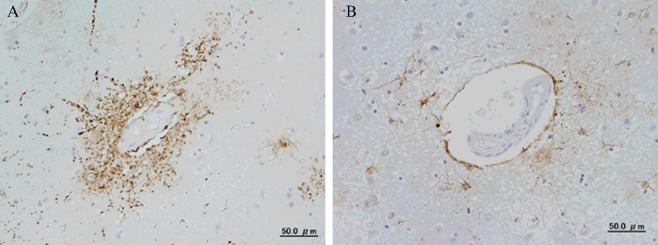
Immunostaining of glial fibrillary acidic protein (GFAP) in the grey matter of parietal cerebral cortex. (A) Acute methamphetamine fatality (blood MA concentration: 2.71 μmol/dL, 45-year-old man, 28 h postmortem); (B) fire fatality (blood carboxyhemoglobin concentration: 78.5%, 65-year-old man, 18 h postmortem).

**Figure 3.**
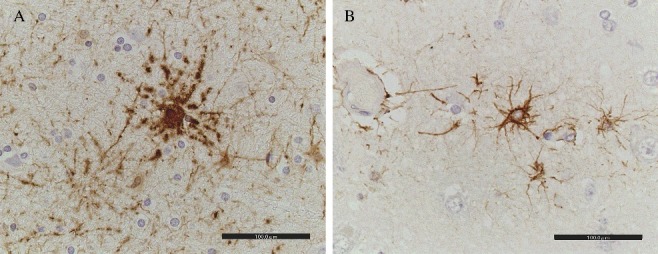
Beaded processes of astrocytes in the grey matter of parietal cerebral cortex. (A) Acute methamphetamine fatality (blood MA concentration: 2.71 μmol/dL, 45-year-old man, 28 h postmortem); (B) fire fatality (blood carboxyhemoglobin concentration: 78.5%, 65-year-old man, 18 h postmortem).

GFAP immunohistochemistry demonstrated that hypertrophic astrocytes could be seen in layers IV, V, and VI in the parietal cerebral cortex of MA abusers (Figure 1), and this result could not be clearly obtained by HE staining. In the grey matter, GFAP-positive astrocytes showed increased size of the astrocytes surrounding capillaries (Figure 2) and clasmatodendrosis (hypertrophic cell bodies and beaded processes) (Figure 3).

**Figure 4.**
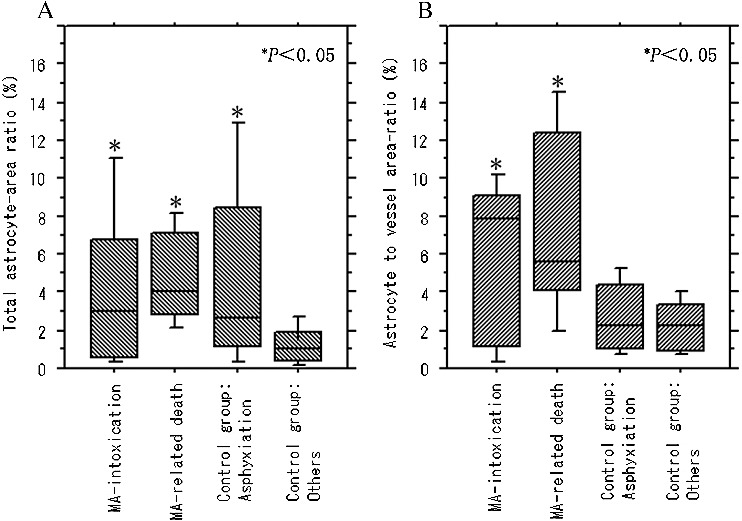
Comparison of (A) total astrocyte-area ratio (%) and (B) astrocyte-to-vessel area ratio (%) in the grey matter between MA-detected patients and control cases.

Quantitative analyses showed a significantly higher value of the total astrocyte area ratio in the grey matter in acute MA intoxication (0.29%–12.83%; median, 2.97%) and other causes of death in MA-related death (1.77%–8.93%; median, 4.05%) compared with the non-MA-detected control group including acute injury death, fire fatalities and myocardial infarction (0.04%–4.83%; median, 1.05%), except for asphyxiation (0.23%–13.58%; median, 2.62%) (Figure 4(A)). The astrocyte-to-capillary vessel area ratio in the grey matter was significantly higher in acute MA intoxication (0.29%–13.78%; median, 7.86%) and other causes of death in MA-related death (1.77%–8.93%; median, 4.05%) than in non-MA-detected control groups including asphyxiation (0.74%–5.40%; median, 2.28%) and other control groups (0.36%–5.42%; median, 2.27%) (Figure 4(B)). There was no significant difference between subgroups of acute injury death, fire fatalities and myocardial infarction (*P* > 0.05). Total capillary vessel area did not show any significant difference between the causes of death.

**Figure. 5:**
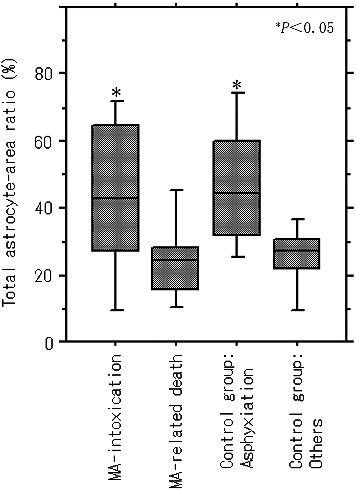
Total astrocyte-area ratio (%) in the white matter with regard to the cause of death.

In the white matter, the total astrocyte area ratio was significantly higher in acute MA intoxication (4.55%–82.72%; median, 42.48%) and asphyxia (24.30%–77.34%; median, 44.53%) than in the other groups involving MA-related death (9.09%–47.51%; median, 24.53%) and the non-MA-detected control group including acute injury death, fire fatalities and myocardial infarction (1.89%–40.54%; median, 27.02%) (Figure 5). There was no significant difference between the subgroups of acute injury death, fire fatalities and myocardial infarction (*P* > 0.05). The increases in the values mentioned earlier did not show any relationship with blood MA level, age, sex, survival or postmortem time.

## Discussion

In the present study, hypertrophic changes in the astrocytes were diffusely seen in the grey matter of the cerebral cortex in MA abusers (acute MA fatality intoxication and other causes of deaths in MA abusers); however, this finding was not evident in the white matter for the other causes of death, except for asphyxiation, among MA abusers and non-abusers. This type of diffuse hypertrophy or swelling of astrocytes in the white matter may have been caused by factors that were not specific to MA abuse. However, clasmatodendrosis of astrocytes accompanied by an increase in the astrocyte-to-vessel area ratio, showing a change of astrocytes at the blood brain barrier (BBB), was observed in the grey matter of the cerebral cortex in both acute MA fatality and other causes of death in MA abusers, whereas it was not seen in non-abusers.

Clasmatodendrosis is an irreversible astroglial degenerative change, which includes extensive swelling and vacuolization of cell bodies in addition to disintegrated and beaded processes [30]. *In vitro* and *in vivo* models of chronic MA abuse showed not only an increase in the number of astrocytes [31–33] but also a dose-dependent change in the activation of astrocytes in response to oxidative stress caused by toxic doses of MA [34–36], which can be associated with neuroprotective cellular events and increased tolerance.

The above observations suggest that chronic MA abuse can be a factor that induces hypertrophy of astrocytes in the grey matter of the cerebral cortex in humans, possibly involving an oxidative stress response associated with increased tolerance [31–33,37–39], although diffuse astrocytic swelling may also occur in acute MA intoxication and asphyxiation. In acute fatal MA intoxication, non-specific astrocytic swelling may additionally be induced, consequently showing diffuse enlargement of astrocytes in the white matter, which is similar to that in asphyxial death. Moreover, beaded processes of GFAP-positive astrocytes can be induced in the grey matter by acute MA abuse, as a possible result of acidosis and energy failure caused by hypoxic-ischemic damage [40,41] or lysosome-derived autophagic astroglial death [30].

## Conclusion

The present study suggests that an increase in the number and hypertrophic changes of astrocytes in the grey matter in MA abusers can be the outcome of long-term abuse, while disintegrating distal processes may exist only in acute fatal MA intoxication.
